# Harnessing alternative sources of antimicrobial resistance data to support surveillance in low-resource settings

**DOI:** 10.1093/jac/dky487

**Published:** 2018-12-13

**Authors:** Elizabeth A Ashley, Nandini Shetty, Jean Patel, Rogier van Doorn, Direk Limmathurotsakul, Nicholas A Feasey, Iruka N Okeke, Sharon J Peacock

**Affiliations:** 1 Myanmar–Oxford Clinical Research Unit (MOCRU), Yangon, Myanmar; 2 Centre for Tropical Medicine and Global Health, University of Oxford, Oxford, UK; 3 National Infection Service, Public Health England, 61 Colindale Avenue, Colindale, London, UK; 4 Centers for Disease Control and Prevention, Atlanta, GA, USA; 5 Centre for Tropical Medicine, Oxford University Clinical Research Unit, Ha Noi, Vietnam; 6 Mahidol-Oxford Tropical Medicine Research Unit, Faculty of Tropical Medicine, Mahidol University, Bangkok, Thailand; 7 The Liverpool School of Tropical Medicine, Liverpool, UK; 8 Malawi Liverpool Wellcome Trust Clinical Research Programme, Blantyre, Malawi; 9 Department of Pharmaceutical Microbiology, Faculty of Pharmacy, University of Ibadan, Ibadan, Nigeria; 10 London School of Hygiene and Tropical Medicine, London, UK

## Abstract

One of the most pressing challenges facing the global surveillance of antimicrobial resistance (AMR) is the generation, sharing, systematic analysis and dissemination of data in low-resource settings. Numerous agencies and initiatives are working to support the development of globally distributed microbiology capacity, but the routine generation of a sustainable flow of reliable data will take time to establish before it can deliver a clinical and public health impact. By contrast, there are a large number of pharma- and academia-led initiatives that have generated a wealth of data on AMR and drug-resistant infections in low-resource settings, together with high-volume data generation by private laboratories. Here, we explore how untapped sources of data could provide a short-term solution that bridges the gap between now and the time when routine surveillance capacity will have been established and how this could continue to support surveillance efforts in the future. We discuss the benefits and limitations of data generated by these sources, the mechanisms and barriers to making this accessible and how academia and pharma might support the development of laboratory and analytical capacity. We provide key actions that will be required to harness these data, including: a mapping exercise; creating mechanisms for data sharing; use of data to support national action plans; facilitating access to and use of data by the WHO Global Antimicrobial Resistance Surveillance System; and innovation in data capture, analysis and sharing.

## Introduction

Surveillance is central to understanding the global burden of antimicrobial resistance (AMR). The generation of surveillance data begins with appropriate sampling of patients with a suspected infectious disease (diagnostic stewardship). Surveillance of sepsis is one example of this, although other specimen types will be required for more comprehensive surveillance. Culture and antimicrobial susceptibility testing of pathogens can improve individual patient management through optimization of drug therapy and supports the appropriate use of drugs (antibiotic stewardship). These data are commonly collated to inform local empirical prescribing policies for patients presenting with infectious disease syndromes. National data may then be collected by ministries of health for the purposes of surveillance, establishing evidence-based guidelines, creating programmes of prevention and resource planning. Finally, national data may be submitted to global surveillance initiatives, which are used to document and track rates of resistance over time, signal where and when interventions are needed and identify countries that require support to build capacity. The most prominent global initiative for the surveillance of bacterial pathogens (excluding TB) is the WHO Global Antimicrobial Resistance Surveillance System (GLASS),^1^ which collects and reports data on resistance rates aggregated by country.

This description of the generation, flow and analysis of AMR data represents an ideal situation in which locally generated microbiological results move from a patient care setting to a national or supranational network, but the reality is that these data are fragmented and dispersed. A recent review commissioned by the Fleming Fund created an inventory of supranational AMR surveillance networks in low- and middle-income countries (LMICs) between January 2000 and August 2017.^2^^,^^3^ This identified 72 supranational networks for AMR surveillance of bacteria, fungi, HIV, TB and malaria, of which 34 are ongoing.^2^^,^^3^ Their median duration was 6 years (range 1–70 years) and the median number of LMICs included in each network was 8 (range 1–67). This scenario is not limited to the lowest-resource settings. A review of European AMR surveillance found similar fragmentation and heterogeneity, with numerous local and national systems that lacked coordination, harmonization and information-sharing with international networks.^4^ There was also inadequate standardization of epidemiological definitions, samples and data collected, microbiological testing methods and data-sharing policies.^4^

**Figure 1.**
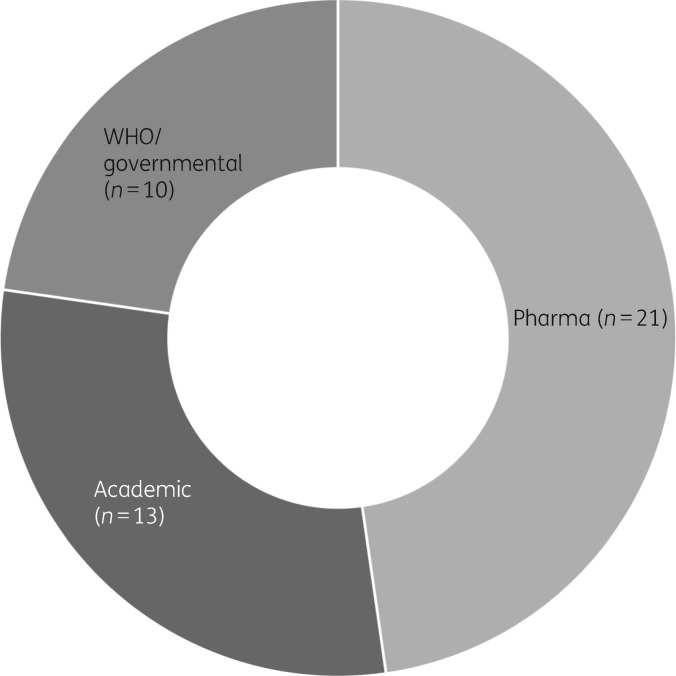
AMR surveillance networks since 2000. Sunburst chart representing 44 supranational networks performing AMR surveillance in bacteria (not including TB) categorized according to their lead organization type (pharmaceutical industry, academia, WHO/governmental). Adapted from reference ^1^.

Categorization of the 72 LMIC networks identified in the review revealed that the minority (*n *=* *26) were led by governments or the WHO, with the remainder being associated with academia (*n *=* *24) or pharma (*n *=* *22).^2^^,^^3^ The number of networks that provided unrestricted access to the data was low (*n *=* *3); the remainder were closed (no access) (*n *=* *38) or categorized as ‘shared or unclear’ (*n *=* *31; shared meaning that data sharing is restricted to specific groups or consortium members). The proportion of networks identified for bacteria (excluding TB) classified by the type of network provider is reproduced in Figure 1. Although this does not equate to the proportion of data generated by each network, it is notable that for bacterial pathogens the majority of networks are led by researchers and pharma. This represents a substantial untapped source of data from settings where the need for surveillance data is greatest and could provide a short-term solution that bridges the gap between now and the time when routine surveillance capacity will have been established.

The purpose of this article is to explore how such information generated in countries with weak AMR surveillance systems could be harnessed for patient and public health, including consideration of the strengths and weaknesses of these data, mechanisms to increase their standardization, harmonization and sharing, and the benefits that could be derived by investing in innovation.

## Alternative sources of AMR data generated in LMICs

### Pharma

Pharmaceutical companies generate a large volume of high-quality bacterial susceptibility data before and after new agents are marketed, in order to fulfil regulatory requirements. These data are largely undisclosed, but some companies are now providing aggregated data, including Pfizer, which has developed ATLAS (Antimicrobial Testing Leadership and Surveillance), a searchable database on resistance to Pfizer anti-infective agents.^5^ The SENTRY Antimicrobial Surveillance Program is a notable commercial surveillance programme run by JMI Laboratories since 1997 that collects data from >200 sites worldwide,^6^ largely from the USA and Europe. Findings based on aggregated data of specific species or genera are published, but the dataset is proprietary and not available for wider use. GSK began the Survey of Antibiotic Resistance (SOAR) study in 2002. This focuses on the effectiveness of antibiotics in the treatment of community-acquired respiratory tract infections.^7^ SOAR concentrates in particular on countries and regions for which little other susceptibility data are available; the findings are regularly reported in the published literature.^8^^,^^9^

Pharma data have several strengths. Isolates are obtained from a global distribution. Organism identification and susceptibility testing procedures are strictly standardized and quality controlled according to international standards. Isolates are transported and retested in a central accredited laboratory, ensuring reliability and reproducibility of results. However, there are several notable disadvantages. There are often no metadata (clinical presentation and outcomes or demographic information) associated with the isolates. Organism sampling fulfils the requirements of the particular pharma project rather than being representative or generalizable to the local population and centralized testing means that quality-controlled test data are not available to guide individual patient care; also, local laboratories do not benefit from improved quality management as a result of participation. Locations perceived to represent small markets are typically under-represented. Furthermore, there is no information on denominator data and so the results may be biased and may not reflect the true burden of resistance in the area tested.

### Academia

Researchers generate a wealth of data on AMR in numerous LMICs. The reasons for data generation include the study of infectious disease aetiology and associated antimicrobial susceptibility, therapeutic drug trials and studies on disease pathogenesis and the molecular biology of bacteria. Some research laboratories have become embedded within district hospitals or other healthcare facilities in LMICs where they provide the only source of ongoing diagnostic microbiology, adopting a model in which research and the provision of microbiological services work in partnership. Compared with pharma data, academic data often provide greater depth of information for specific populations (e.g. all patients treated in a particular hospital) and many have been operating for several years or even decades.

Research data have several potential strengths. Laboratories may be subject to good laboratory practice when the information generated is performed to specific standards, supported by methods that are rigorously evaluated through robust quality-assurance and quality-control programmes. The methods used to generate bacterial susceptibility data are notoriously error prone and data generated outside of quality-controlled laboratories may be of suboptimal quality;^10^^,^^11^ the inclusion of quality-assured research data in national and global databases could increase the proportion of accurate data points. Patient information may also be collected on clinical presentation, duration of hospital stay, antibiotic treatment, complications and outcome. Furthermore, understanding the trajectory for resistance often requires evaluation of susceptibility data over long timescales and newer national surveillance programmes may not yet have sufficient retrospective information to make the most of new data.

Researcher-defined infectious disease aetiology and common susceptibility patterns, even if determined intermittently, support empirical prescribing in settings where the treatment of febrile illness is based on clinical features and there is no funding to offer routine testing to patients. Empirical prescribing may result in overuse of antibiotics and increased rates of resistance. In the longer term, the ideal would be to have a global surveillance programme that promotes laboratory testing for better patient care and directed antibiotic therapy associated with antibiotic stewardship. In the short term, however, empirical prescribing is an essential approach that saves lives, provided that data are sufficiently contemporaneous. Although not always the case, research-driven AMR data may not be generated with sufficient speed to provide information that guides individual patient treatment.

When research laboratories are embedded in district hospitals in rural areas (as they often are) inclusion of their data in global databases can also go some way to balancing the selection bias that can arise. For example, WHO GLASS requires participating countries to establish at least one surveillance site and then extend the number over time. In LMICs where diagnostic microbiology laboratories are scarce, these are most likely to be situated in tertiary hospitals. Bacteria isolated at tertiary hospitals in any part of the world are more likely to be associated with patients with more severe or complex disease, patients who have received numerous courses of antibiotics, patients with prolonged hospitalization and those transferred from other hospitals with hospital-acquired infection, all of which will lead to over-representation of bacterial resistance compared with patients in district hospitals or in the wider community.

Research-generated data also have several potential limitations. Data may be biased, including ascertainment and sampling biases.^12^ For example, the study design may target patient subsets that do not reflect the wider population with infectious diseases, such as sampling of patients within cohorts that have better access to care or patients with the most severe infection syndromes. Since a proportion of bloodstream infections will be hospital acquired, studies of severe invasive disease may inflate rates of resistance and may not capture milder forms of community-associated infection in patients treated as outpatients, which may be caused by organisms with lower AMR. Research data may also include duplicate samples. Six main types of potential bias that may influence the validity or interpretation of surveillance data have been identified and these provide a framework for reviewing the use of research data in AMR surveillance (use of inadequate or inappropriate denominator data; case definitions; case ascertainment; sampling bias; failure to deal with multiple occurrences; and biases related to laboratory practice and procedures).^13^

### Private laboratories

A source of susceptibility data that may remain unseen by national surveillance systems, particularly in many LMIC settings, are laboratories in private hospitals that generate data for patient care.^14–16^ The quality of data generated by private laboratories varies considerably, but those that are accredited and perform quality-assured services produce data of similar or better quality than that produced by public laboratories. In India, almost all medical laboratories accredited by the National Accreditation Board for Testing and Calibration Laboratories (NABL) are in the private sector and, in South Africa, >80% of South African National Accreditation System (SANAS)-accredited medical laboratories are in the private sector.^15^ This has led to calls to utilize these data and the inclusion of such data by initiatives such as ResistanceMap.^17^ This displays AMR data on 12 bacterial species isolated in 49 countries, collected between 1999 to 2015 (depending on the country), together with antibiotic consumption data from 75 countries between 2000 and 2014. The primary sources of data are public and private laboratory networks that routinely collect susceptibility results, but data from India come exclusively from the private sector.

Private laboratories can provide extensive datasets for populations for whom there is a very limited supply of reliable AMR data from alternative sources, but again can suffer from the types of bias already described for research data. Furthermore, these laboratories often serve a subset of more affluent people, including medical tourists and members of the expatriate community, which may not provide an accurate representation of rates of resistance for similar types of infection in the wider population.

## Barriers to using alternative sources of AMR data from LMICs

Despite the obvious utility of placing AMR surveillance data generated by academia, pharma or private laboratories into the public domain, very little of these data generated in LMICs is utilized by organizations involved in regional or global surveillance. There are several barriers that prevent this from happening. Data are held in numerous silos with highly restricted access. Academics generate data that usually remain private until published in peer-reviewed journals; individual patient-level data may not be released or may be delayed by several years from the point of collection because of the time taken to analyse, write and publish. Pharma companies have to jump through several legal hoops before they release their data into the public domain. Even if researchers and pharma companies are keen to deposit data towards global analyses, data aggregation is hampered by a lack of harmonization in data collection, a lack of tools that allow easy data deposition and the lack of a framework that prevents publication of their data by unscrupulous competitors. Furthermore, GLASS collects and reports data on resistance rates aggregated at national level by ministries of health and cannot currently accept information generated by research activities or pharma. In general, national programmes take ownership of in-country surveillance activities and agreement may not be reached for direct data deposition to WHO GLASS by non-governmental groups. Furthermore, AMR surveillance data can represent potentially sensitive data, particularly when these describe high rates of resistance or the emergence of a novel resistance mechanism to a key antibiotic.

## Mechanisms to unlock AMR data

### Incentivizing access to data from academia and pharma

‘Bottom-up’ research and pharma activity that generates AMR data is not public health surveillance in the strict sense. Furthermore, the majority of researchers and pharma-employed scientists would be quick to highlight that public health surveillance is neither their responsibility nor area of interest. Agreeing on the principle that researchers and pharma companies could make a major contribution to global surveillance should be aligned with the recognition that this is not their primary purpose and will be associated with a financial cost. Debate is required about incentives to support the additional workload associated with sharing data with national programmes or other repositories and who should coordinate this. This discussion could draw on experience gained from academic incentives during the development of the WorldWide Antimalarial Resistance Network (WWARN) platform.^18^ Any investment should not detract from funding that provides improved data sources for patient care, surveillance and prevention of AMR.

The flow of research-generated data into global initiatives could be facilitated by funders, who could develop guidelines on sharing of specific datasets, a procedure that could become an integral component of a successful funding award. This is already the case for some forms of data, examples being the submission of all sequence data generated by the Wellcome Sanger Institute to public databases and funding by Wellcome being linked to an open access publication policy. Such changes would require a clear plan for formatting and destination of data deposition. Journals and publishers could also develop guidelines on data deposition for publications on drug-resistant infection and could make this a necessary part of submission. Data released into public databases by researchers would need to be protected by data access committees or through other mechanisms, but this is not insurmountable because solutions are already in place for numerous types of data. There are also examples of training and data sharing/open access agreements having been developed that are contextualized and locally acceptable.^19^^,^^20^

Wellcome has begun to address access to untapped sources of global surveillance data held by pharma through a recently funded project conducted by the Open Data Institute. This has created a mechanism to bring together leaders from public health and the pharmaceutical industry, who are collaborating to explore how value could be added by the reuse of available data. An evaluation of the mechanisms and barriers to making this open access has been completed and detailed in a post-project report.^21^ One proposal is to create a public–private partnership to improve local laboratory capacity. Another is to suggest that relevant metadata and denominator data are also collected, fulfilling the objectives of the pharma project whilst also providing information about local AMR prevalence that, while not informing individual patient care, could inform empirical prescribing protocols.

### Supporting ministries of health to access data

Ministries of health could collaborate with research institutions where this is not already the case or a public–private partnership could be forged so that data generated by research, pharma or private laboratories can be submitted to GLASS as national data. There are examples of research units in Asia and Africa that have already developed close and sustainable working relationships with the relevant ministry of health, which uses the information provided to shape national prescribing policies. In this way, countries can be empowered to utilize data generated in their own territory, with local researchers undertaking in-depth analyses using a range of sources, thereby encouraging comparisons of incidence and prevalence rates of drug-resistant infection between different areas in the country in order to monitor the disease burden and the impact of action plans in each area. This also represents an important training opportunity for government staff, who can develop the technical capability to analyse data that are ultimately generated through country-led capacity building.

### The need for specialist networks

Having argued that the development of new initiatives that effectively replicate WHO GLASS and that fragment data are generally to be discouraged, there are some circumstances when additional networks add vital new information. A notable example is the Institute for Health Metrics and Evaluation, which has recently been funded by a joint award from Wellcome, the UK Fleming Fund and the Bill and Melinda Gates Foundation to gather, map and analyse disease and death caused by drug-resistant infections. This will be used to quantify the global burden of disease (GBD) due to drug-resistant infections compared with other diseases and causes of death, and so inform policy- and decision-making. Estimating the GBD due to AMR faces numerous challenges, including difficulties in linking surveillance data with clinical or outcome data and causal attribution, and cannot be regarded as a routine surveillance activity at present.

## Investing in innovation

Investment is needed to promote innovation in AMR surveillance. For example, harnessing emerging technologies relating to ‘Big Data’ and artificial intelligence could lead to more effective mechanisms of AMR data capture, sharing and analysis tools. An innovative system to support automatic data harmonization between different laboratories and institutions could achieve numerous objectives, including: an inbuilt system to standardize data analysis and quality tests for data from multiple sources; capture of patient outcome data to underpin calculations of GBD; and rapid relay of information to treating clinicians, e.g. via electronic decision support algorithms. Data could be automatically linked to national agencies and international data repositories. Innovation in data capture would benefit from early involvement of experts in the social sciences so that the behaviour change required to support buy-in is an integral part of planning and development. Mapping of data sources may also require consideration of the regulatory environment in some settings. Innovation needs to be linked to effective translation, scale-up and integration, and assessed in terms of impact on policy.^22^ Any alternative system developed will need to be either fully interoperable with WHO GLASS or able to generate data in a format that can be uploaded.

## Conclusions and next steps

Our understanding of the GBD due to drug-resistant infection in LMICs is rudimentary and data from academia, pharma and private laboratories could make an important and rapid contribution. A dialogue is required to determine how data generated in LMICs might flow from these bodies to national and global AMR surveillance networks and how they might support the development of laboratory and analytical capacity, including robust quality-management systems, for prospective data collection. This should build on current initiatives such as the Fleming Fund, which is providing regional grants to collect existing AMR and antimicrobial use data from all possible sources. Table 1 summarizes proposed changes that could help to bring this into effect.

**Table 1.** Key actions to harness AMR data from alternative sources in LMICs

Objectives	Actions
Map data	Map and evaluate quality/utility of data held and generated Determine how to enhance these resources, i.e. through the addition of patient outcome data Determine how data can contribute to the measurement of the GBD due to AMR
Create mechanisms for data sharing and capacity building	Identify incentives that promote the contribution of data from academic, pharma and private laboratories Agree the basis for data sharing, including ownership, ethical and legal considerations Develop mechanisms for data harmonization, collation and analysis Promote private–public partnerships to build capacity in local laboratories for patient care and surveillance
Facilitate update of data nationally and internationally	Use data to support national action plans Seek mechanisms and create funding opportunities to support uptake of academia/pharma/private laboratory data by WHO GLASS and other data-sharing initiatives
Innovation in data capture, analysis and sharing	Create a data collection interface that supports: Case-based surveillance Quality assurance and control and a universal reporting standard for patient data Automated linkage to national agencies and international data repositories

## Funding

SEDRIC is supported by the Wellcome Drug Resistant Infection Priority Programme. The funder had no involvement in the content, writing or submission of the paper.

## Transparency declarations

None to declare.
